# *Staphylococcus aureus* and* Escherichia coli* in Curd Cheese Sold in the Northeastern Region of South America

**DOI:** 10.1155/2017/8173741

**Published:** 2017-12-06

**Authors:** Tamiles Barreto de Deus, Ludmilla Santana Soares Barros, Ricardo Mendes da Silva, Wanessa Karine da Silva Lima, Danuza das Virgens Lima, Adriana dos Santos Silva

**Affiliations:** ^1^Universidade Federal do Recôncavo da Bahia, Centro de Ciências Agrárias, Ambientais e Biológicas, Rua Rui Barbosa, No. 710, Campus Universitário, 44380-000 Cruz das Almas, BA, Brazil; ^2^Universidade Federal do Recôncavo da Bahia, Centro de Ciências da Saúde, Santo Antônio de Jesus, BA, Brazil

## Abstract

The present study evaluated the microbiological and sanitary quality of curd cheese sold on the beaches of the Itaparica Island, Brazil, and verified whether a correlation exists between the commercialization conditions and the microbiological data. The research was performed between December 2015 and March 2017. Sixty samples of rennet-containing cheese were collected to estimate the populations of mesophylls, psychrotrophic microorganisms, mold and yeast,* Staphylococcus aureus*, total coliforms, and* Escherichia coli*. An observational analysis was performed during the collection, using a checklist to verify the sellers' sanitary conditions and cheese marketing. A high nonconformity index was registered regarding aspects in the checklist. In the microbiological analyses, the number of mesophylls in raw and roasted samples ranged from 7,88 to 14,82 log CFU/mL, and those of psychrotrophs ranged from 2,80 to 3,84 log CFU/mL. Meanwhile, mold and yeast levels in the samples ranged from 8,06 to 5,54 log CFU/mL,* S. aureus *was detected at levels from 3,24 to 4,94 log CFU/mL, and the total coliform counts ranged from 4,48 to 7,18 log CFU/mL. The number of* E. coli *specimens ranged from 2,96 to 5,75 log CFU/mL. Microbial insecurity was noted for commercialized curd cheese, and the need for intervention was indicated.

## 1. Introduction

Street-vended food is highly popular in many countries, feeding several people and providing a decent income for families [1]; however, it is a major cause of food-transmitted diseases (FTDs) and various health issues [2]. 

Traditional street-vended food could represent a major risk to public health, due to the unsanitary and unhygienic conditions, including poor infrastructure, improper storage temperature, and poor hygiene among the handlers during commercialization [3]. Curd cheese is one of the most commercialized and consumed foods even though it is homemade and the absence of microbiological contamination cannot be ensured; however, it has social and economic relevance, especially for small-sized farms [4].

Informal food vending is a common practice characterized by high microbiological risk, carrying serious health liabilities for consumers [5].

Moreover, beaches represent the preferred commercialization site, resulting in high contamination rates [6]. Considering the informal sale of milk and its derivatives, several studies previously identified the main pathogenic microorganisms, namely,* Staphylococcus aureus*,* Salmonella *spp.,* Listeria monocytogenes,* and* Escherichia coli* [7–10].

Knowledge concerning the microbiological quality of commercialized curd cheese is valuable because the consumption of contaminated food may cause FTDs, thus, representing a public health problem. The present study evaluated the microbiological and sanitary quality of curd cheese sold on the beaches of the Itaparica Island and verified whether a relationship exists between the commercialization conditions and the reported microbiological data.

## 2. Materials and Methods

The study was undertaken at 10 beaches on the Itaparica Island. The following beaches were chosen at random: Praia de Mar Grande, Ponta de Areia, Conceição, Caixa Prego, Barra do Gil, Aratuba, Coroa, Barra do Pote, Barra Grande, and Berlinque.

Sixty samples of curd cheese were collected. Three curd cheese vendors, chosen at random, provided three raw and three roasted pieces of cheese. Samples were collected in the daytime on Saturdays and Sundays between December 2015 and March 2016, during holidays, when majority of food makers and vendors were available.

Samples were collected aseptically, transported in an isothermal container with ice, and maintained under refrigeration until microbiological analyses were conducted at the Microbiology and Animal Parasitology laboratory of the Universidade Federal do Recôncavo da Bahia (UFRB), Brazil.

An observational analysis was performed at the time of sample harvesting using a checklist based on Decree 216/2004 and Normative Instruction 30/2001, which included questions on the handlers' hygiene and sanitary habits and the commercialization and storage of cheese. The temperature of cheese was registered immediately after retrieving the samples to verify compliance with the legislation [11, 12].

The microbiological analyses comprised the total counts of psychrotrophic microorganisms, mesophylls, mold and yeast,* S. aureus, *total coliforms, and* E. coli.*

The pour plate technique was employed for the microbiological analyses of psychrotrophic microorganisms and mesophylls, with plate count agar used as the culture medium. Samples (25 mg in total) were obtained from several sites of each specimen and placed in 225-mL sterile flasks containing 0.1% peptonized water, and 1 mL of each dilution was transferred to a Petri plate with 25 mL of previously heated agar at 43–45°C.

After homogenization and solidification, plates were incubated in a buffer at 7°C for 10 days or at 35°C for 48 h for psychrotrophic microorganisms and mesophylls, respectively. Colonies were counted using a colony counter. The average colony number on each plate was multiplied by the corresponding dilution factor, and the result was presented as log UFC/mL.

The spread plate method was employed to calculate the mold and yeast counts in Sabouraud dextrose agar medium. Plates containing 25 mL of culture medium were prepared and inoculated with 0.1 mL of each dilution on the medium surface, and the inoculum was spread carefully. Plates were then incubated in BOD buffer at 24°C for 48–72 h [13].

Coliforms were counted using Chromocult® coliforms agar, following the manufacturer's instructions. Colonies were counted using a colony counter, in particular, dark blue to violet colonies were classified as* Escherichia coli*, and salmon red colored colonies as other coliforms.

*S. aureus *levels were analyzed using a rapid method with Petrifilm™ plates (3M Company), following the manufacturer's instructions. Red-violet colonies were identified as* S. aureus.*

Statistical analysis was performed using SPSS 17, and descriptive analysis comprised means and standard deviations for quantitative variables and proportions for qualitative variables. Student's* t*-test was employed for independent samples to compare the microorganism levels between raw and roasted cheese. *p* ≤ 0.05 denoted statistical significance.

Checklist variables according to mesophyll, psychrotroph, mold and yeast,* S. aureus, *total coliform, and* E. coli *levels in raw and roasted curd cheese were analyzed using Student's* t*-test.

The relationship between temperature and microorganism levels was assessed using Pearson's correlation analysis. *p* ≤ 0.01 denoted statistical significance.

## 3. Results

### 3.1. The Workers

Table 1 shows the results of observational analysis of food handlers' hygiene, which is important for preventing food contamination. This analysis revealed a low index for personal care, as 90, 13.3, and 53.3% of handlers had long and dirty fingernails, used adornments, and wore a beard, respectively.

### 3.2. Microbiological Profile of Raw and Roasted Curd Cheese

Table 2 presents the results of microbiological analyses of raw and roasted curd cheese. A comparison of the average microbial content of cheese samples using Student's* t*-test revealed that the levels of mesophylls, mold, yeast,* S. aureus*, total coliforms, and* E. coli* were lower for roasted cheese (*p* < 0.5), indicating high levels of contamination of raw cheese.

The levels of mesophyll microorganisms in raw and roasted cheese ranged from 7.88 to 14.82 log CFU/g. When compared to the mesophyll microorganisms, psychrotrophic microorganisms (Table 2) were less prevalent in raw (3.64 log CFU/g) and roasted (2.80 log CFU/g) cheese. Curd cheese samples displayed high mold and yeast levels, averaging 8.06 log CFU/g in raw cheese and 5.54 log CFU/g in roasted cheese (Table 2).

### 3.3. Relationship between Temperature and Microorganism Levels

Table 3 reveals the negative correlations between the temperature of roasted cheese and the levels of mesophylls,* S. aureus*, and* E. coli*; in particular, higher roasting temperatures were associated with lower microorganism levels. Although the initial population counts decreased, the samples exhibited minimum, mean, and maximum temperatures of 44.0, 58.2, and 72.4°C, respectively.

## 4. Discussion

### 4.1. The Workers

Furlaneto-Maia et al. [14] and Chukuezi [15] also reported high noncompliance rates among the food handlers concerning their body care. Inadequacy concerning these factors contributes to increased transmission risks of pathogenic agents via commercialized food.

All food vendors were observed to handle money and food simultaneously, without washing their hands, jeopardizing proper food handling (Table 1). Cortese et al. [16] also reported low efficiency for this factor, confirming global studies regarding the lack of hygiene among the food handlers. Microorganisms are present on the hands in substantial amount, and they are extremely difficult to eliminate. Thus, proper hygiene is vital for preventing FTDs.

When the food handlers were examined for their uniforms (Table 1), all were found without gloves, headwear, uniforms, and closed shoes; this was contrary to RDC 216, which insists that all food handlers should wear clean and good uniforms compatible with their activities.

The commercialized cheese had a wide range of hole sizes (Table 1), which may be related to poor conditions during manufacturing. Lactose-fermenting bacteria also produce CO_2_, and they may give rise to microcavities known as cheese eyelets [17].

No commercialized cheese samples had pungent odors or surface slime, and no inadequacies were registered (Table 1). Meanwhile, Franco and Landgraf [18] found that these items are highly efficient concerning the aspects of hygiene and food safety.

All commercialized cheese was stored at room temperature even though the technical rules on the identity and quality of curd cheese stipulate that cheese should be stored at a temperature not exceeding 12°C during conservation and commercialization [11].

Storage data revealed carelessness regarding the containers in which the food was preserved. In fact, 73.3% (Table 1) of containers did not meet the cleanliness standards. In their study on the hygienic and sanitary conditions of food sellers in Uberaba, Brazil, Souza et al. [19] reported a lack of compliance regarding the same items. This lack of compliance may increase the risk of food contamination given the direct link between storage conditions and contamination.

The Table 2 has values which exceeded the values reported by Meneses et al. [20], who recorded levels of 8.1 and 6.4 log CFU/g for samples of raw and roasted curd cheese, respectively.

High mesophyll levels (Table 2) were also reported by Delamare et al. [21] in their analysis of homemade Serrano cheese samples manufactured in Brazil. Specifically, the counts of mesophyll bacteria ranged from 7.91 to 9.47 log CFU/g. Although no normative standards exist at present, estimates of such populations are relevant because high levels of mesophyll microorganisms in food indicate deficient hygienic and sanitary conditions.

Although the Brazilian law has not established a limit for psychrotrophic bacteria, Chen et al. [22] stressed that these microorganisms are responsible for the deterioration of milk and its derivatives and that they may alter milk and its derivatives by producing enzymes that hydrolyze proteins and lipids, making them inappropriate for consumption.

Although curd cheese is a typical Brazilian product and because mold and yeast can deteriorate the quality of dairy products, Brazilian law has not established the maximum levels for these microorganisms in cheese.

Silva et al. [23] analyzed the curd cheese manufactured from raw and pasteurized milk in three dairies in the backlands of the state of Alagoas, Brazil, and reported levels of 4.58, 4.66, and 4.86 log CFU/g, respectively. Perin et al. [24] analyzed homemade Minas cheese and detected average mold and yeast levels of approximately 5 log CFU/g.

Total coliform levels averaged 7.18 and 4.48 log CFU/g in raw and roasted cheese samples, respectively. Salotti et al. [25] reported that the levels of these microorganisms in food identify the product's sanitary and conservation state; thus, they are indicative of consumers' health risks.

Brazilian legislation establishes a limit of 2.7 log CFU/g for* E. coli*, whereas the levels in the present analysis revealed 5.75 log CFU/g in raw cheese and 2.96 log CFU/g in roasted cheese. The presence and high levels of microorganisms were also reported by Oliveira et al. [26], who assessed the microbiological quality of curd cheese in the municipality of Cabo de Santo Agostinho, PR, Brazil and verified that 80.95% of samples had bacterial levels exceeding the standard limits.

High levels of* E. coli *exceeding the standard limits define the product as inappropriate for commercialization and consequently for human consumption due to the fecal contamination [27].

The examined cheese samples had high* S. aureus *levels, namely, 4.94 log CFU/g in raw samples and 3.24 log CFU/g in roasted samples. The results illustrated that* S. aureus *levels in cheese exceeded the standard limits and evidenced serious microbiological liabilities in the commercialized curd cheese.

Tigre and Borelly [28] also measured* S. aureus *counts in curd cheese commercialized by the street sellers on the Itapuã Beach in Salvador, BA, Brazil, during morning and afternoon, with the results ranging from 4.84 to 5.73 log CFU/g in the morning and 4.57 to 6.36 log CFU/g in the afternoon.

*S. aureus *levels exceeding the standard recommendations (2.7 log CFU/g) may be related to the contamination of prime matter during manufacturing. This fact may be also linked to the handlers because pathogens have common interaction mechanisms with the host and they may be frequently found in skin and mucus [29].

*S. aureus *levels were high in a study by Machado et al. [30], who detected levels exceeding 8 log CFU/g in curd cheese from several dairies.* S. aureus* produces enterotoxins, which cause food intoxication; thus, they pose a health risk to consumers [31].

### 4.2. Relationship between Temperature and Microorganism Levels

According to ABERC [32], most samples failed to reach the temperature recommended for roasted food, namely, a minimum temperature of 74°C at the geometric center, or time and temperature combinations such as 65°C/15 min or 70°C/2 min.

In the case of raw cheese, there was a weak negative correlation between* E. coli *levels and temperature (Table 3). Statistical analysis evidenced the opposite correlation. Specialized literature revealed that the best conditions for the growth of these microorganisms are between 30 and 45°C [18].

The temperature of raw cheese exceeded the standard limit [11], with minimum, mean, and maximum temperatures of 20.6, 31.3, and 35.1°C, respectively. These findings reveal carelessness regarding the handling procedures, with serious risks for consumers.

### 4.3. Relationship between the Hygienic and Sanitary Conditions in Commercialization and Microorganism Levels

There was no significant difference in the levels of microorganisms related to variables such as storage site and the use of adornments and wearing of a beard by the handlers. Despite these findings, when these aspects are inadequate, they positively affect the food contamination.

On the contrary, a statistically significant correlation (*p* < 0.05) was seen between the total coliform levels and compliance among the handlers regarding clean nails. In fact, total coliform levels were higher among noncompliant handlers. Contamination of food handlers' hands is one of the factors that most strongly contributes to the risk of FTDs.

The microbiological results underscore that data from the checklist may be directly related to improper hygiene-sanitary conditions and prove that these aspects are the main causes of contamination.

## 5. Conclusion

High levels of deteriorating and pathogenic microorganisms revealed poor hygienic and sanitary quality in the products analyzed and the need for good practices in food manipulation and commercialization, coupled with efficient monitoring and surveillance by authorities. Based on the established Brazilian microbiological standards, most raw and roasted cheese samples were classified as unfit for human consumption.

## Figures and Tables

**Table 1:** Results for items assessed using the checklist related to the hygiene and sanitary habits of the handlers and the manner of curd cheese commercialization on the beaches of the Itaparica Island, BA, Brazil, 2016.

	% noncompliant	% compliant
*Assessed items referring to handlers*		
Short and clean fingernails lacking nail polish?	90	10
Were adornments used?	13.3	86.7
Did males have a beard or mustache?	53.3	46.7
Was the tiller distinct from the food handler?	100	
Did handlers wash their hands prior to food preparation?	100	
Did they wear uniforms?	100	
Did they use headwear?	100	
Was the white uniform clean?	100	
Did they wear closed shoes?	100	
Did they wear gloves during food preparation?	100	
*Assessed commercialization items*		
Were there any eyelets?	100	
Was there any surface slime?		100
Did the cheese smell?		100
Was cheese conditioned in a thermal box at 12°C?	100	
Was the storage area clean?	73.3	26.7

**Table 2:** Results for populations of microorganisms (log CFU/g) in samples of curd cheese sold on the beaches of the Itaparica Island, BA, Brazil, 2016.

Microorganisms	Raw cheese				Roasted cheese				RDC
	log CFU/g				log CFU/g				12/2001
	Min	Max	Av	SD	Min	Max	Av	SD	CRU
Mesophylls	6.33	17.55	14.82	2.92	5.47	10.30	7.88	<1	NA
Psychrotrophs	<1	8.20	3.64	1.78	<1	6.46	2.80	1.58	NA
Mold	<1	12.13	8.06	2.16	<1	9.11	5.54	2.18	NA
*Staphylococcus aureus*	<1	10.41	4.94	2.62	<1	5.41	3.24	1.57	2.7
Total coliforms	4.33	11.02	7.18	1.89	<1	6.79	4.48	1.67	NA
*Escherichia Coli*	<1	10.14	5.75	2.82	<1	5.90	2.96	1.87	2.7

Av: average; Min: minimum; MAX: maximum; SD: standard deviation; NA: not applicable.

**Table 3:** Pearson's correlation analysis between temperature and microorganism levels in raw and roasted curd cheese commercialized on the beaches of the Itaparica Island, BA, Brazil, 2016.

Microorganisms	Correlation coefficient for roasted cheese	Correlation coefficient for raw cheese
Mesophylls	−0.64^*∗*^	0.20
Psychrotrophs	0.13	0.07
Mold	−0.29	−0.29
*Staphylococcus aureus*	−0.39^*∗*^	−0.18
Total coliforms	−0.31	−0.03
*Escherichia coli*	−0.58^*∗*^	−0.55^*∗*^

^*∗*^Statistically significant (*p* < 0.01).
